# An evaluation of methods for the isolation of nontuberculous mycobacteria from patients with cystic fibrosis, bronchiectasis and patients assessed for lung transplantation

**DOI:** 10.1186/s12890-019-0781-2

**Published:** 2019-01-21

**Authors:** D. Stephenson, A. Perry, M. R. Appleby, D. Lee, J. Davison, A. Johnston, A. L. Jones, A. Nelson, S. J. Bourke, M. F. Thomas, A. De Soyza, J. L. Lordan, J. Lumb, A. E. Robb, J. R. Samuel, K. E. Walton, J. D. Perry

**Affiliations:** 1 Microbiology Department, Freeman Hospital, Newcastle upon Tyne, NE7 7DN UK; 2 Faculty of Health and Life Sciences, Northumbria University, Newcastle upon Tyne, UK; 3 Adult Bronchiectasis Service, Freeman Hospital, Newcastle upon Tyne, UK; 4 Adult Cystic Fibrosis Centre, Royal Victoria Infirmary, Newcastle upon Tyne, UK; 5 Paediatric Respiratory Unit, Great North Children’s Hospital, Newcastle upon Tyne, UK; 6 Cardiopulmonary Transplant Service, Freeman Hospital, Newcastle upon Tyne, UK

**Keywords:** Nontuberculous mycobacteria, *Mycobacterium abscessus* complex, *Mycobacterium avium* complex, Cystic fibrosis, Bronchiectasis, Lung transplantation, AFB culture

## Abstract

**Background:**

RGM medium is an agar-based, selective culture medium designed for the isolation of nontuberculous mycobacteria (NTM) from the sputum of patients with cystic fibrosis (CF). We evaluated RGM medium for the detection of NTM in patients with CF (405 samples), bronchiectasis (323 samples) and other lung diseases necessitating lung transplantation (274 samples).

**Methods:**

In total, 1002 respiratory samples from 676 patients were included in the study. Direct culture on RGM medium, with incubation at two temperatures (30 °C and 37 °C), was compared with conventional culture of decontaminated samples for acid-fast bacilli (AFB) using both a solid medium (Löwenstein-Jensen medium) and a liquid medium (the Mycobacterial Growth Indicator Tube; MGIT).

**Results:**

For all three patient groups, significantly more isolates of NTM were recovered using RGM medium incubated at 30 °C than by any other method (sensitivity: 94.6% vs. 22.4% for conventional AFB culture; *P* < 0.0001). Significantly more isolates of *Mycobacterium abscessus* complex were isolated on RGM at 30 °C than by AFB culture (sensitivity: 96.1% vs. 58.8%; *P* < 0.0001). The recovery of *Mycobacterium avium* complex was also greater using RGM medium at 30 °C compared to AFB culture (sensitivity: 83% vs. 70.2%), although this difference was not statistically significant and a combination of methods was necessary for optimal recovery (*P* = 0.21).

**Conclusions:**

In the largest study of RGM medium to date, we reaffirm its utility for isolation of NTM from patients with CF. Furthermore; we show that it also provides an effective tool for culture of respiratory samples from patients with bronchiectasis and other lung diseases.

## Background

Nontuberculous mycobacteria (NTM) are recognized as significant respiratory pathogens, particularly in individuals with pre-existing lung disease, for example, patients with cystic fibrosis (CF) [1]. In the largest studies of individuals with CF, the prevalence of NTM in sputum has been estimated at 6–13% [2] and is reported to be increasing [3–6]. The dominant pathogens among NTM causing respiratory disease in CF include members of the *Mycobacterium abscessus* complex (MABSC) and *Mycobacterium avium* complex (MAC). Chronic infection with MABSC is particularly problematic in CF and is associated with a significant decline in lung function [7]. It can also be extremely difficult to eradicate due largely to high levels of intrinsic antimicrobial resistance. NTM are also important pathogens in patients with non-CF bronchiectasis and their prevalence appears to be similar to that found in CF, with one meta-analysis reporting a prevalence of 9.3% [8], however most studies show a clear dominance of MAC [9].

Reduced lung function associated with CF or other lung pathologies may necessitate lung transplantation as a treatment of last resort. However, infection with NTM may complicate lung transplantation, with severe post-operative infections frequently associated with MABSC [10–13].

The isolation of NTM from the respiratory tract may reflect innocuous colonization or active infection characterized by progressive inflammatory lung damage [1]. NTM-pulmonary disease (NTM-PD) is defined by the presence of specific microbiological, clinical and radiological features [14]. Therefore, although isolation of NTM by culture does not provide a diagnosis for NTM-PD, culture remains a cornerstone of diagnosis [15]. The recommended procedure for isolation of NTM is conventional acid-fast bacilli (AFB) culture that utilizes methods originally designed for isolation of *Mycobacterium tuberculosis*. These include the decontamination of sputum samples (e.g. using alkali or acid) followed by culture for up to 8 weeks using both solid and liquid media. One limitation is that the decontamination process is known to reduce the viability of NTM thus reducing the sensitivity of culture methods [1, 16, 17].

In 2016, Preece et al. reported the development of RGM (“Rapidly-growing mycobacteria”) medium; a selective agar that allows isolation of NTM without decontamination of samples [18]. The medium was primarily designed for recovery of rapidly-growing mycobacteria (e.g. MABSC) and consequently a 10-day incubation period was initially recommended. In a comparison with *Burkholderia cepacia* selective agar, RGM medium showed a clear superiority for recovery of NTM (sensitivity 98% vs. 31%; *P* < 0.0001) [18]. RGM medium subsequently showed equivalent sensitivity to the Mycobacterial Growth Indicator Tube (MGIT), an automated liquid culture system, for the detection of total NTM from 187 patients with CF [19]. To accommodate the isolation of MAC and other slow-growing species, Plongla et al. extended the incubation of RGM to 28 days and compared its performance to conventional methods that included inoculation of MGIT plus Löwenstein-Jensen medium (LJ). In an evaluation with 212 respiratory samples from CF patients, a much higher yield of NTM was recovered using RGM medium including a significantly higher yield of MABSC [20].

The aim of this study was to compare 28-day culture on RGM medium to conventional AFB culture using MGIT and LJ with respiratory samples from three patient groups: (1) patients with CF, (2) patients with non-CF bronchiectasis (hereafter referred to as bronchiectasis; Br), and (3) patients assessed for lung transplantation with a variety of other lung diseases. It is well recognised that different species of NTM have different temperature preferences and that duplicate sets of media at two incubation temperatures are frequently recommended for optimal recovery of a wide range of species [14]. We therefore inoculated duplicate plates of RGM medium and compared incubation at 30 °C with incubation at 37 °C.

## Methods

### Patient samples

A total of 1002 respiratory samples were routinely collected from 676 patients for conventional AFB culture over a 16-month period between April 2017 and July 2018. Samples were submitted at clinics dedicated to the management of CF (*n* = 405) and Br (*n* = 323) or from patients attending the cardiothoracic centre for lung transplantation due to other lung diseases (*n* = 274). The latter group were all assessed for lung transplantation with most patients (91%) sampled pre-transplantation and a minority of patients (9%) sampled in the post-transplantation period. We cultured all consecutively submitted samples from these patient groups with no other selection criteria. Most samples were cultured on the day of sample collection and the remainder after storage for up to 48 h at 4 °C. A full description of the samples and patients is available in Table 1.

**Table 1:** Summary of basic patient demographics and types of sample included in the study

	All	Cystic Fibrosis	Bronchiectasis	Other lung diseases*
No. of patients	676	279	239	158
Assessed for lung Tx	218	66	8	144
Post lung Tx	29	14	1	14
Mean age (years)	47	28	63	56
Age range (years)	2–91	2–77	9–91	13–72
% Male	48	52	38	56
% Female	52	48	62	44
No. of samples	1002	405	323	274
Sputum	969	401	312	256
BAL	22	3	4	15
Bronchial secretions	10	0	7	3
Tracheal aspirate	1	1	0	0
*Patients (*n* = 158) with other lung diseases included those with the following conditions:				
			*n*	
Chronic obstructive pulmonary disease			51	
Unspecified interstitial lung disease			28	
Idiopathic pulmonary fibrosis			25	
Fibrotic lung disease			22	
Pulmonary hypertension			6	
Sarcoidosis			6	
Nonspecific interstitial pneumonia			5	
Hypersensitivity pneumonitis			4	
Lymphangioleiomyomatosis (LAM)			4	
Complex congenital lung disease			1	
Graft versus host disease			1	
Histiocytosis X			1	
Langerhans cell histiocytosis			1	
Obliterative bronchiolitis			1	
Pleuroparenchymal fibroelastosis (PPFE)			1	
Progressive rheumatoid associated lung disease			1	

Abbreviations: *lung Tx* lung transplantation, *BAL* broncho-alveolar lavage

### Culture on RGM medium

Batches of RGM medium were prepared in-house as previously described [21]. All culture procedures were carried out in a Class I safety cabinet. Samples were treated with an equal volume of Sputasol (Oxoid; SR0233A) and then mixed by vortexing at intervals until visibly homogeneous, except for samples of broncho-alveolar lavage (BAL) that did not require homogenization. Aliquots of 100 μL were inoculated onto two plates of RGM medium. Each plate was placed in a sealed plastic bag and incubated for 28 days: one plate at 30 °C and one plate at 37 °C. Plates were examined for growth after 4 days, 7 days and then weekly. BAL samples were centrifuged at 3000 rpm for 10 min and the deposit was re-suspended in 1 mL of supernatant and cultured as described above.

Any colonies recovered on RGM medium were subjected to a Gram stain and a stain for AFB using the auramine-phenol method. Non-mycobacteria were identified to species level using matrix-assisted laser desorption/ionization time-of-flight mass spectrometry (MALDI-TOF MS, Bruker). Suspected mycobacteria were also subjected to MALDI-TOF MS, following the extraction protocol recommended by Bruker (the Mycobacteria Extraction (MycoEX) Method). Species identification was achieved by matching spectra to those available in the Bruker Mycobacteria Library version 4.0 (bead method). Any suspected strains of mycobacteria that could not be identified using MALDI-TOF MS (or required further sub-speciation e.g. for MAC) were referred to the National Mycobacterium Reference Service in Birmingham, UK, for whole genome sequencing. Isolates confirmed as MABSC by MALDI-TOF MS were referred to the Antimicrobial Resistance and Healthcare Associated Infections Reference Unit, Public Health England, (PHE, Colindale, UK) for sub-speciation by sequencing of at least two of three housekeeping genes (*rpoB*, *hsp65* and *sodA*) as previously described [22].

### AFB culture

The methods used were selected from those recommended by Public Health England [23]. All procedures were carried out in a Class I safety cabinet within a category 3 containment facility. Samples that had been homogenized with Sputasol (see above) were centrifuged at 3000 g for 15 min and the supernatant removed. A thin smear of the deposit was used to prepare a slide for staining using the auramine-phenol method and subsequent examination for AFB using fluorescence microscopy. All respiratory samples were subjected to decontamination using the modified Petroff method prior to culture for AFB. A 7 mL aliquot of sodium hydroxide solution (4% *w*/*v*) was added to the centrifuged deposit and vigorously mixed using a vortex mixer after 0, 5, 10, 15 and 20 min. After a total of 25 min, the sample was neutralised using 14 mL phosphate buffer with pH indicator (final pH 6.8). The sample was re-centrifuged and the deposit re-suspended in 2 mL of phosphate-buffered saline (PBS) at pH 6.8. A 0.5 mL aliquot was then inoculated into a MGIT and loaded onto the automated instrument for a minimum of 28 days incubation. A further 0.2 mL was inoculated onto a pyruvate LJ slope and this was incubated for 8 weeks at 36 °C. For CF samples only, a further 0.2 mL was inoculated onto a pyruvate LJ slope and this was incubated for 4 weeks at 30 °C. For certain samples, the incubation of MGITs was extended to 56 days. This extended incubation was prompted by various factors including contamination of LJ slope(s), previous history of mycobacterial infection or a positive AFB smear.

For CF samples only, an additional round of decontamination was carried out in parallel using half of the original centrifuged deposit. An equal volume of sulfuric acid (0.5 N) was added to the deposit, mixed by vortexing and left for 60 min at ambient temperature. Approximately 20 mL of sterile distilled water was then added and the sample centrifuged at 3000 g for 15 min. A 2 mL aliquot of PBS (pH 6.8) was then added to the deposit. A 0.5 mL aliquot was then inoculated into a MGIT and loaded onto the automated instrument for a minimum of 28 days incubation. Aliquots of 0.2 mL were then taken for inoculation of two pyruvate LJ slopes (one incubated at 30 °C for 4 weeks and one incubated at 36 °C for 8 weeks). All of the culture methods are summarised in Table 2.

**Table 2:** Summary of culture methods

	Culture media	Decontamination	Inoculum	Incubation	Reading times
All samples	RGM at 30 °C	None	0.1 mL homogenized sample*	4 weeks	4,7,14,21,28 days
	RGM at 37 °C	None	0.1 mL homogenized sample*	4 weeks	4,7,14,21,28 days
CF samples	MGIT	H_2_SO_4_	0.5 mL of deposit.	≥ 4 weeks	Continuous
	MGIT	NaOH	0.5 mL of deposit.	≥ 4 weeks	Continuous
	LJ at 30 °C	H_2_SO_4_	0.2 mL of deposit.	4 weeks	Weekly
	LJ at 30 °C	NaOH	0.2 mL of deposit.	4 weeks	Weekly
	LJ at 36 °C	H_2_SO_4_	0.2 mL of deposit.	8 weeks	Weekly
	LJ at 36 °C	NaOH	0.2 mL of deposit.	8 weeks	Weekly
Non-CF samples	MGIT	NaOH	0.5 mL of deposit.	≥ 4 weeks	Continuous
	LJ at 36 °C	NaOH	0.2 mL of deposit.	8 weeks	Weekly

*Broncho-alveolar lavage (BAL) samples were concentrated by centrifugation prior to culture on RGM. Abbreviations: *MGIT* Mycobacterial Growth Indicator Tube, *LJ* Löwenstein-Jensen medium, H_2_SO_4_; 0.5 N sulfuric acid, NaOH; 4% (*w*/*v*) sodium hydroxide

For a positive MGIT, the entire content was decanted into a sterile bottle and centrifuged at 3000 rpm for 15 min. The culture deposit was re-suspended in 1 ml PBS with 4–6 glass beads, vortexed and left to settle for 5 min. A thin smear was then prepared for staining for AFB and a chocolate agar plate was inoculated and incubated in a sealed bag at 36 °C for 24 h. For samples yielding non-mycobacteria, if the original sample was < 14 days old, the process of decontamination and culture in a fresh MGIT was repeated. For samples not yielding any microorganisms, the remainder of the deposit was inoculated into fresh culture medium and the bottle re-loaded on the MGIT instrument. LJ slopes were examined weekly and any colonies of suspect mycobacteria were subjected to staining for AFB and also subcultured on a chocolate agar plate as described above. Samples that could be excluded as mycobacteria based on colony characteristics and a negative AFB smear were not processed further. Suspect isolates of mycobacteria were referred to the National Mycobacterium Reference Service in Birmingham, UK, for whole genome sequencing. Isolates of *M. abscessus* complex were referred to PHE, Colindale, UK, for sub-speciation as described above.

Any isolate of NTM that was only recovered by RGM at one incubation temperature (or was only isolated using AFB culture) was re-inoculated onto two plates of RGM medium and two plates of RGM medium without antibiotic supplement that were incubated at 30 °C and 37 °C for up to 28 days. This was performed by obtaining a fresh culture of each strain on Middlebrook agar and preparing a 0.5 McFarland suspension in sterile saline (0.85%). A 1 μL aliquot was then spread onto each plate. Information obtained from these subcultures was not used in the calculation of specificity or sensitivity for the various culture methods.

### Statistical analysis

RGM culture at 30 °C was compared with RGM culture at 37 °C using McNemar’s test with the continuity correction applied. Statistical significance was taken as *P* <  0.05. The same method was used to compare RGM culture at 30 °C with conventional AFB culture.

## Results

### Optimal temperature for incubation of RGM medium

Table 3 shows the numbers of NTM that were recovered using RGM at two different temperatures. Significantly more isolates of NTM were recovered on RGM medium at 30 °C (*n* = 334) than at 37 °C (*n* = 79) (*P* < 0.0001). This was mostly due to the isolation of species of doubtful clinical significance (e.g. *Mycobacterium gordonae*) that did not grow at 37 °C. However, for pathogenic species known to be causes of lung infections, there was also a greater yield at 30 °C. For example, the number of isolates of MABSC was significantly higher at 30 °C than at 37 °C (*P* = 0.016) and the yield of MAC was also higher (*P* = 0.27).

**Table 3:** Nontuberculous mycobacteria isolated from 1002 respiratory samples using RGM medium at two incubation temperatures

	Total RGM	RGM	RGM
		30 °C	37 °C
*M. chelonae* complex	86	86	2
*M. paragordonae / gordonae*	56	56	0
*M. llatzerense*	52	52	0
*M. abscessus* complex (MABSC)	51	49	39
*M. avium* complex (MAC)	43	39	34
*Mycobacterium* species	27	26	1
*M. mucogenicum-phocaicum* group	8	8	0
*M. septicum*	6	6	0
*M. fortuitum*	2	2	1
*M. lentiflavum*	2	2	0
*M. obuense*	2	2	0
*M. paraffinicum*	2	2	0
*M. arupense*	1	1	0
*M. mageritense*	1	1	1
*M. neoaurum*	1	1	0
*M. simiae*	1	1	1
Total	341	334	79

For the few isolates of MABSC and MAC that were only recovered at 37 °C, only 1–3 colonies were recovered suggesting that the failure to recover these isolates at 30 °C was most probably due to chance, rather than a preference for a higher incubation temperature (all isolates of MAC and MABSC grew well when re-inoculated onto RGM and incubated at 30 °C). Overall, the data suggested little added benefit of using RGM at 37 °C.

### Comparison of culture using RGM medium at 30 °C with conventional AFB culture

As we concluded that incubation of RGM medium at 37 °C offered little additional benefit, we then performed a detailed comparison of the other two methods i.e. RGM medium at 30 °C (“RGM 30 °C”) versus AFB culture. Table 4 summarises all of the NTM isolated from 1002 respiratory samples and comparative data for these two methods. A total of 353 isolates of NTM were recovered using a combination of all methods and these were isolated from 281 samples (i.e. 28% of samples yielded NTM). A total of 57 samples (5.7%) contained more than one species of NTM (with one specimen containing as many as four distinct species). Mixtures of NTM species were isolated from 50 samples by just using RGM 30 °C whereas only one mixture of NTM was detected by just using AFB culture.

**Table 4:** Nontuberculous mycobacteria recovered from 1002 respiratory samples from 676 patients by RGM at 30 °C and conventional AFB culture

	Total^a^	RGM 30 °C	AFB Culture	RGM 30 °C	AFB Culture	
	*n*	*n*	*n*	Sensitivity (%)^b^		***P*** ^***b***^
*M. abscessus* complex (MABSC)^c^	51	49	30	96.1	58.8	< 0.0001
*M. avium* complex (MAC)^d^	47	39	33	83	70.2	0.21
*M. chelonae* complex^e^	86	86	4	100	4.7	< 0.0001
*M. paragordonae / gordonae*	57	56	3	98.2	5.3	< 0.0001
*M. llatzerense*	52	52	0	100	0	< 0.0001
*Mycobacterium* species	27	26	0	96.3	0	< 0.0001
*M. mucogenicum-phocaicum* group	8	8	0	–	–	–
*M. septicum*	6	6	0	–	–	–
*M. simiae*	5	1	5	–	–	–
*M. fortuitum*	2	2	1	–	–	–
*M. lentiflavum*	2	2	0	–	–	–
*M. obuense*	2	2	0	–	–	–
*M. paraffinicum*	2	2	0	–	–	–
*M. arupense*	1	1	0	–	–	–
*M. iranicum*	1	0	1	–	–	–
*M. kumamotonense*	1	0	1	–	–	–
*M. mageritense*	1	1	0	–	–	–
*M. neoaurum*	1	1	0	–	–	–
*M. xenopi*	1	0	1	–	–	–
Total	353	334	79	94.6	22.4	< 0.0001

^a^MABSC (*n* = 2), MAC (*n* = 3), and *Mycobacterium* sp. (*n* = 1) are included in the totals but were only isolated using RGM medium at 37 °C

^b^Sensitivity and probability (*P*) were calculated only where the number of isolates (*n*) was > 10

^c^MABSC included subspecies *abscessus* (*n* = 30), subspecies *massiliense* (*n* = 13) and 8 isolates unresolved to subspecies level

^d^MAC included subspecies *avium* (*n* = 16), subspecies *intracellulare*-*chimaera* (*n* = 27) and *M. colombiense* (*n* = 1). 3 isolates could not be resolved further than *M. avium* complex

^e^*M. chelonae* complex includes *M. chelonae* / *salmoniphilum* (*n* = 77) and *M. franklinii* (*n* = 9)

Of the 353 isolates, 334 (94.6%) were recovered on RGM 30 °C compared with 79 (22.4%) recovered by AFB culture (*P* < 0.0001). Notably, the sensitivity of RGM 30 °C was significantly higher than AFB culture for detection of MABSC (sensitivity 96.1% vs. 58.8%; *P* < 0.0001). The number of MAC isolates recovered on RGM was also higher than the number recovered by AFB culture, but this was not statistically significant (sensitivity 83% vs. 70.2%; *P* = 0.21) and eight strains of MAC remained undetected using RGM 30 °C. All of these eight strains grew well when re-inoculated onto RGM and incubated at 30 °C.

Acid-fast bacilli were observed by microscopy in only 24 respiratory samples (2.4%). For two of these samples, no NTM (or any other type of AFB) were recovered by any of the culture methods. The remaining 22 smear-positive samples were accounted for by growth of MABSC (*n* = 12), MAC (*n* = 6) and single isolates of *Mycobacterium chelonae*, *M. gordonae*, *Mycobacterium simiae* and *Mycobacterium* species. All except the *M. simiae* isolate were recovered on RGM 30 °C and all except the single isolate of *Mycobacterium* species were recovered by AFB culture. *M. tuberculosis* is rarely encountered in these patient groups in our centre, and no strains of this species were recovered from any samples in this study.

In every patient group, large numbers of NTM belonging to species other than MABSC and MAC were isolated using RGM 30 °C and most of these remained undetected using AFB culture. In most cases, these species were isolated on RGM medium with very low colony-counts. For example, *M. llatzerense* was isolated from 52 samples but the mean colony count on RGM 30 °C was only 3.7 colonies, with only one colony of *M. llatzerense* recovered from half of these 52 samples. In contrast, the average colony count for MABSC was 67 on RGM 30 °C.

MABSC was detected in samples from 18 patients using RGM 30 °C where AFB cultures were negative. In 10 of these patients (55%), the same subspecies was documented in at least one additional sample taken from the same patient on a separate occasion. For seven patients, subsequent sputum cultures were negative when cultures were repeated and for one patient repeat samples were not submitted. MAC was detected in samples from 10 patients using RGM where AFB cultures were negative. In seven of these patients (70%), the same subspecies was documented in at least one additional sample taken from the same patient on a separate occasion. For two patients, subsequent sputum cultures were negative when cultures were repeated and for one patient repeat samples were not submitted.

The *M. chelonae* complex consists of *M. chelonae* and three additional validated species; *Mycobacterium franklinii*, *Mycobacterium immunogenum* and *Mycobacterium salmoniphilum* [24, 25]. *M. chelonae* complex was detected in samples from 64 patients using RGM where AFB cultures were negative. For 37 of these patients, repeat samples were cultured and *M. chelonae* complex was recovered from 14 patients (38%).

When *M. llatzerense* or *M. gordonae/paragordonae* were isolated, culture of repeat samples was much less likely to yield the same species. For example, *M. llatzerense* was detected in samples from 50 patients using RGM where AFB cultures were negative. For 33 of these patients, repeat samples were cultured and *M. llatzerense* was recovered from only three patients (9.1%). Similarly, for *M. gordonae/paragordonae* only 2/31 patients (6.4%) who had repeat cultures yielded the same species.

For 63/1002 specimens (6.3%), AFB cultures (both solid and liquid media) were found to be overgrown by species other than mycobacteria resulting in the premature termination of cultures, despite repeat attempts at decontamination. This contamination rate was highest for CF samples (14%) when compared with Br (1.2%) and others (0.7%). In comparison, non-mycobacteria were recovered from 11.2% of samples on RGM 30 °C (21.4% for CF, 6.5% for Br and 1.5% for others). However, in the vast majority of cases, the growth of non-mycobacteria on RGM plates was restricted and did not compromise isolation of NTM. The most common non-mycobacteria recovered on RGM 30 °C were Gram-negative bacilli including *Achromobacter* species and *Burkholderia cepacia* complex (data not shown).

The mean time to detection for NTM was similar for AFB culture and RGM 30 °C, despite the fact that MGIT was subjected to continuous monitoring and RGM was only examined after 4 days and then weekly. For MABSC, the mean time to detection was 5.5 days for AFB culture (range: 2–21 days) and 6.1 days for RGM 30 °C (range: 4–28 days). For MAC the mean time to detection was 14.6 days (range: 4–56 days) for AFB culture and 15.2 days for RGM 30 °C (range: 4–28 days).

### Cystic fibrosis group

MABSC was recovered from 35 specimens that were submitted from 31 patients with CF indicating that 11.1% of patients with CF were infected or colonized by MABSC. A total of 26 isolates of MAC were recovered from 21 patients with CF (prevalence: 7.5%). The number of MABSC recovered from patients with CF was significantly higher using RGM 30 °C [*P* = 0.004] with almost one third of isolates undetected by AFB culture (Table 5). Consequently, MABSC remained undetected in 10 patients using AFB culture alone compared with a failure to isolate MABSC in only one patient using RGM 30 °C. Both methods performed equally well for recovery of MAC (*P* = 1.00). One isolate of *Mycobacterium iranicum* did not grow on RGM medium at either temperature when re-inoculated as a pure strain and incubated for 28 days.

**Table 5:** Nontuberculous mycobacteria recovered from 405 respiratory samples from 279 patients with cystic fibrosis

	Total^a^	RGM 30 °C	AFB Culture	RGM 30 °C	AFB Culture	
	*n*	*n*	*n*	Sensitivity (%)^b^		*P* ^*b*^
*M. abscessus* complex (MABSC)	35	34	24	97.1	68.6	0.004
*M. avium* complex (MAC)	26	20	19	76.9	73.1	1.000
*M. chelonae* complex	22	22	2	100	9.1	< 0.0001
*M. llatzerense*	14	14	0	100	0	0.0005
*M. paragordonae / gordonae*	11	10	2	90.9	18.2	0.027
*Mycobacterium* species	11	10	0	90.9	0	0.004
*M. mucogenicum-phocaicum* group	3	3	0	–	–	–
*M. paraffinicum*	2	2	0	–	–	–
*M. septicum*	2	2	0	–	–	–
*M. arupense*	1	1	0	–	–	–
*M. fortuitum*	1	1	1	–	–	–
*M. iranicum*	1	0	1	–	–	–
*M. obuense*	1	1	0	–	–	–
Total	130	120	49	92.3	37.7	< 0.0001

^a^MABSC (*n* = 1), MAC (*n* = 2), and *Mycobacterium* sp. (*n* = 1) are included in the totals but were only isolated using RGM medium at 37 °C

^b^Sensitivity and probability (*P*) were calculated only where the number of isolates (*n*) was > 10

### Bronchiectasis group

A total of 15 isolates of MAC were recovered from specimens submitted by 12 patients from the overall cohort of 239 patients with Br giving a prevalence of 5%. Eight isolates of MABSC were detected from seven patients with Br (prevalence: 2.9%), with MABSC detected in 6/7 patients using RGM 30 °C and in only 1/7 patients using AFB culture. As shown in Table 6, large numbers of other NTM species were isolated using RGM 30 °C, most of which were not recovered using AFB culture.

**Table 6:** Nontuberculous mycobacteria recovered from 323 respiratory samples from 239 patients with bronchiectasis

	Total ^a^	RGM 30 °C	AFB Culture	RGM 30 °C	AFB Culture	
	*n*	*n*	*n*	Sensitivity (%)^b^		*P* ^*b*^
*M. abscessus* complex (MABSC)	8	7	2	–	–	–
*M. avium* complex (MAC)	15	14	12	93.3	80	0.62
*M. chelonae* complex	32	32	0	100	0	< 0.0001
*M. paragordonae / gordonae*	32	32	1	100	3.1	< 0.0001
*M. llatzerense*	25	25	0	100	0	< 0.0001
*Mycobacterium* species	9	9	0	–	–	–
*M. mucogenicum-phocaicum* group	4	4	0	–	–	–
*M. septicum*	3	3	0	–	–	–
*M. kumamotonense*	1	0	1	–	–	–
*M. lentiflavum*	1	1	0	–	–	–
*M. mageritense*	1	1	0	–	–	–
*M. neoaurum*	1	1	0	–	–	–
*M. simiae*	2	1	2	–	–	–
Total	134	130	18	97	13.4	< 0.0001

^a^MABSC (*n* = 1) is included in the totals but was only isolated using RGM medium at 37 °C

^b^Sensitivity and probability (*P*) were calculated only where the number of isolates (*n*) was > 10

### Other lung diseases

MABSC and MAC were isolated sporadically from patients with other lung conditions whose disease severity had prompted an assessment for lung transplantation. For example, MABSC was isolated from eight samples (from five patients) using RGM 30 °C compared with four samples (from 2 patients) using AFB culture. Each of the five patients with MABSC had a different underlying lung disease. Six isolates of MAC were isolated from samples collected from five patients, with MAC detected in four patients using RGM 30 °C and two patients using AFB culture. Of the five patients with MAC, two had chronic obstructive pulmonary disease, two had lymphangioleiomyomatosis (LAM) and one had sarcoidosis.

As shown in Table 7, large numbers of other NTM species were isolated using RGM 30 °C, most of which were not recovered using AFB culture. However, occasional strains could only be isolated using AFB culture. For example, *Mycobacterium simiae* was recovered from three separate sputum samples submitted from a patient with idiopathic pulmonary fibrosis awaiting lung transplantation. None of these isolates was recovered on RGM medium and they did not grow on RGM medium within 28 days when re-inoculated. *M. simiae* was successfully recovered from a patient with Br in this study and was isolated on RGM medium in a previous study [21]. A strain of *Mycobacterium xenopi* was recovered on LJ medium after 8 weeks of incubation at 36 °C but was not recovered by RGM at either temperature (or using MGIT). After re-inoculation, this isolate of *M. xenopi* did not grow on RGM medium at 30 °C but grew well on RGM medium after 21 days incubation at 37 °C.

**Table 7:** Nontuberculous mycobacteria recovered from 274 respiratory samples from 158 patients assessed for lung transplantation with other respiratory diseases

	Total^a^	RGM 30 °C	AFB Culture	RGM 30 °C	AFB Culture	
	*n*	*n*	*n*	Sensitivity (%)^b^		*P* ^*b*^
*M. abscessus* complex (MABSC)	8	8	4	–	–	–
*M. avium* complex (MAC)	6	5	2	–	–	–
*M. chelonae* complex	32	32	2	100	6.3	< 0.0001
*M. paragordonae / gordonae*	14	14	0	100	0	0.0005
*M. llatzerense*	13	13	0	100	0	0.0009
*Mycobacterium* species	7	7	0	–	–	–
*M. simiae*	3	0	3	–	–	–
*M. fortuitum*	1	1	0	–	–	–
*M. lentiflavum*	1	1	0	–	–	–
*M. mucogenicum-phocaicum* group	1	1	0	–	–	–
*M. obuense*	1	1	0	–	–	–
*M. septicum*	1	1	0	–	–	–
*M. xenopi*	1	0	1	–	–	–
Total	89	84	12	94.4	13.5	< 0.0001

^a^MAC (*n* = 1) is included in the total but was only isolated using RGM medium at 37 °C

^b^Sensitivity and probability (*P*) were calculated only where the number of isolates (*n*) was > 10

## Discussion

In 2016, the Cystic Fibrosis Foundation (CFF) and European Cystic Fibrosis Society (ECFS) published consensus recommendations to support and standardise the management of infections caused by NTM in children and adults with CF [1]. The recommended methods to culture respiratory samples for NTM are summarized in Table 8. At the time these recommendations were compiled, in our view, they appeared to reflect the best available evidence. However, there are a number of drawbacks and/or limitations to these recommendations, some of which are recognized in the consensus document. For example, the decontamination of respiratory samples is laborious and, according to the recommendations, may substantially reduce the viability of mycobacteria, thereby reducing sensitivity for detection of NTM [1]. The method is slow, requiring at least 6 weeks before a negative result can be issued (or longer if repeat rounds of decontamination are required). Furthermore, contamination events cannot be completely averted meaning that cultures may have to be abandoned in some cases. In such cases, patients may have to revisit the clinic to resubmit further sample(s). At least two different types of culture media are required as well as sophisticated instrumentation for automated monitoring of liquid cultures. In many laboratories this means that samples need to be processed in a separate location to where routine culture for other bacterial pathogens is performed. Perhaps mindful of the burden that these procedures place on clinical laboratories, routine screening for NTM is only recommended on an annual basis. This could be detrimental to the early detection of NTM colonization / infection and might preclude opportunities for early clinical management, particularly for MABSC. For example, in a recent paper, Ravnholt et al. argue persuasively that the window of opportunity, before MABSC transforms from a mucosal colonizer to a chronic biofilm infection, is where microbial eradication is most likely to be successful, making early diagnosis essential for improved outcomes [26]. Delayed detection might also compromise efforts to limit patient-to-patient spread.

**Table 8:** CFF / ECFS recommendations for microbiological procedures for diagnosis of NTM lung disease in patients with cystic fibrosis (CF) [1]

•Cultures for NTM should be performed annually in spontaneously expectorating individuals with a stable clinical course. •In the absence of clinical features suggestive of NTM pulmonary disease, individuals who are not capable of spontaneously producing sputum do not require screening cultures for NTM. •Culture and smears for acid-fast bacilli from sputum should be used for NTM screening. •Oro-pharyngeal swabs should not be used for NTM screening. •Cultures and smears for acid-fast bacilli from sputum, induced sputum, bronchial washings or broncho-alveolar lavage samples can be used to evaluate individuals with CF suspected to have NTM-pulmonary disease. •Respiratory tract samples should be cultured using both solid and liquid media and incubated for a minimum of six weeks. •Respiratory tract samples should be decontaminated using N-acetyl L-cysteine NALC (0.5%) - NaOH (2%). •If a sample remains contaminated with Gram-negative bacteria after standard decontamination, it should be further treated with either 5% oxalic acid or 1% chlorhexidine. •Non-culture based methods should not be used to detect NTM in respiratory tract samples.	

To address these limitations, the use of a highly selective agar-based medium is an attractive option for isolation of NTM, especially if it enables the culture of respiratory samples without decontamination. A previous study from Chapel Hill, United States, using 212 sputum samples from patients with CF showed that culture using RGM medium for 28 days at 30 °C recovered significantly higher numbers of NTM and a much greater range of NTM species, when compared to formal AFB culture [20]. In this study, we have reproduced these findings with respect to CF and we report similar findings for patients with Br and various other lung diseases. In particular, we show a clear advantage for RGM medium for the isolation of MABSC (sensitivity 96.1% vs. 58.8%; *P* < 0.0001) and an equivalent sensitivity of the two methods for the isolation of MAC (sensitivity 83% vs. 70.2%; *P* = 0.21). In the United Kingdom, and elsewhere in Europe, MABSC is the dominant pathogen among NTM in patients with CF [3, 4] and it has been associated with a higher decline in lung function than any other pathogen associated with CF lung disease [7]. For this reason, we have adopted culture on RGM medium as a first line screening test for all samples submitted from patients with CF. Conventional AFB culture is reserved for patients with a strong clinical suspicion of NTM disease, who have negative cultures using RGM.

MABSC is also an important pathogen in the context of lung transplantation as it can cause serious post-operative complications [10–13]. In a recent survey of transplantation centres, 16 of 21 centres that responded to a questionnaire regarded persistent infection with MABSC as a relative contraindication to lung transplantation [27]. A higher number of respondents (18 of 21) required treatment of such infections pre-transplantation [27]. The use of RGM 30 °C was effective for isolation of MABSC from patients awaiting lung transplantation with 8/8 isolates successfully detected from five patients awaiting transplantation. Only 4/8 isolates (from two patients) were detected using AFB culture. In the case of Br, NTM is also an important pathogen and screening is recommended if there are clinical or radiological signs of disease, or if a patient is due to begin long term macrolide therapy [28]. MAC is generally regarded as the principal pathogen among NTM affecting patients with Br [9]. We believe that the data reported here for the use of RGM medium for screening patients with Br justify further studies in different centres.

The fact that a far greater yield of NTM can be recovered using RGM medium is remarkable when considering the relative inocula used for RGM medium and AFB culture. A 100 μL aliquot of sputum diluted (1:1) in sputasol is inoculated onto RGM medium compared with at least 700 μL of a concentrated deposit that is used for AFB culture. For a 10 mL sputum sample, the theoretical inoculum is 70 x higher for AFB culture (or 180 x higher for a CF sputum sample), assuming there are no losses of NTM during the various centrifugation steps. This provides convincing evidence of the deleterious effect of decontamination on the viability of NTM.

**Fig. 1:**
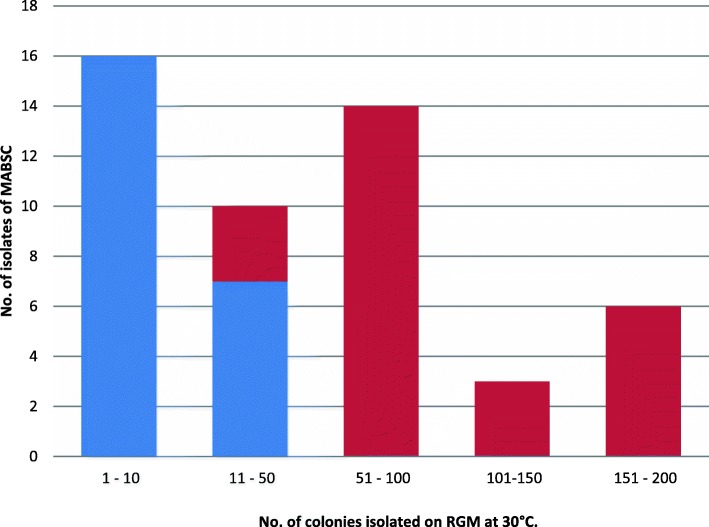
Correlation between colony count of MABSC on RGM at 30 °C and positive AFB culture (red bars) or negative AFB culture (blue bars)

AFB culture is generally able to detect NTM when they are present at high inocula and, in such cases, performance is equivalent to RGM. For example, when the load of NTM was high enough in the sample to result in a positive AFB smear, the sensitivity of the two methods was identical. Furthermore, there was a very clear correlation between the colony count obtained on RGM 30 °C and the likelihood of detection of MABSC by AFB culture. For example, there were 16 specimens with a low colony count (i.e. 1–10 colonies) of MABSC on RGM 30 °C and none of these was detected by AFB culture. Conversely, there were 23 samples that produced a colony count of > 50 MABSC and every one of these was detected by AFB culture (see Fig. 1). A similar trend was observed for MAC (data not shown).

Given that there is a clear association between the failure to detect NTM using AFB culture and lower numbers of NTM in these specimens, a major question relates to the clinical significance of additional isolates of NTM that are only detected using culture on RGM medium. We do not yet have a clear answer to this question. One of the first things to consider when assessing the possibility of NTM-PD versus transient or persistent colonization is whether the same species has been recently isolated from the patient or whether it can be isolated from a subsequent sample. If the presence of NTM is sporadic, it is unlikely to signify NTM-PD. For samples that are positive using RGM 30 °C but negative by AFB culture, we have shown there is a good chance that a repeat sample will give the same result for certain species e.g. for MAC (70%), for MABSC (55%) and, to a lesser extent, *M. chelonae* complex (38%). For other species, such as *M. llatzerense* or *M. gordonae/paragordonae* the number of colonies was typically very low and the chances of isolating the same species from a repeat sample was < 10%. In most if not all cases, the recovery of such species that transiently colonize the respiratory tract as commensals, or arise from environmental contamination, may be regarded as more of a hindrance than a benefit when using RGM medium.

### Limitations of the study

Although the decontamination methods used in this study for AFB culture are consistent with guidelines published in the United Kingdom [23], they are not consistent with the most recent guidelines published by CF societies in 2016 [1]. Furthermore, although all samples were cultured on Löwenstein-Jensen medium for 8 weeks, the duration of incubation of MGIT vials for culture of NTM was typically restricted to 4 weeks due to limitations in the capacity of the MGIT instrument. Although previous studies suggest that incubation of MGIT beyond 4 weeks is very unlikely to increase the yield of NTM [19, 20], we cannot preclude the possibility that more NTM would have been isolated by MGIT if we had incubated all samples for 6 weeks as recommended in the most recent guidelines [1]. Finally, the identification methods used in this study did not generate bacterial names that are consistent with the taxonomic changes to the genus *Mycobacterium* proposed and validated in 2018 [29, 30]. The bacterial names that were valid at the start of this study (in 2017) have been retained for convenience. Consequently, the term ‘NTM’ used in this paper does not just include species of the genus *Mycobacterium* but also species of *Mycobacteroides* (e.g. *Mycobacteroides abscessus*), *Mycolicibacterium* (e.g. *Mycolicibacterium llatzerense*) and *Mycolicibacter* (e.g. *Mycolicibacter kumamotonensis*).

## Conclusions

RGM medium is more sensitive than AFB culture for detection of NTM across multiple patient groups (*P* < 0.0001). RGM should be incubated at 30 °C as originally recommended [18] and an additional plate at 37 °C offers negligible benefit for detection of NTM. RGM also offers a higher sensitivity than AFB culture for detection of MABSC and this relates primarily to the inability of AFB culture to detect low inocula of MABSC. RGM has equivalent sensitivity to AFB culture for the detection of MAC, but optimal detection of MAC can be achieved by combining both methods. Most isolates of MABSC or MAC that are not recovered by AFB culture are recovered from previous or subsequent samples from the same patient indicating a persistent colonisation or infection. A negative consequence of using RGM medium is the isolation of an abundance of species of doubtful clinical significance (e.g. *M.llatzerense* or *M. gordonae*/*paragordonae*), which mostly appear to represent contamination or transient colonization of the respiratory tract.

## Abbreviations

AFB: Acid fast bacilli
BAL: Broncho-alveolar lavage
Br: Non-cystic fibrosis bronchiectasis
CF: Cystic fibrosis
H_2_SO_4_: 0.5 normal (N) sulfuric acid
LJ: Löwenstein-Jensen medium
Lung Tx: Lung transplantation
MABSC: *Mycobacterium abscessus* complex
MAC: *Mycobacterium avium* complex
MALDI-TOF MS: Matrix-assisted laser desorption/ionization time-of-flight mass spectrometry
MGIT: Mycobacterial growth indicator tube
NaOH: 4% weight/volume (*w*/*v*) sodium hydroxide
NTM: Nontuberculous mycobacteria
NTM-PD: Nontuberculous mycobacterial pulmonary disease
PBS: Phosphate-buffered saline
RGM 30 °C: RGM medium incubated at 30 °C for 28 days
